# Sex and HIV Differences in Preserved Ratio Impaired Spirometry (PRISm) Among Ugandans Postpneumonia

**DOI:** 10.1093/ofid/ofae033

**Published:** 2024-01-22

**Authors:** Rebecca A Abelman, Jessica Fitzpatrick, Katerina L Byanova, Josephine Zawedde, Ingvar Sanyu, Patrick Byanyima, Emmanuel Musisi, Jenny Hsieh, Michelle Zhang, Jake Branchini, Abdul Sessolo, Peter W Hunt, Rejani Lalitha, J Lucian Davis, Kristina Crothers, William Worodria, Laurence Huang

**Affiliations:** Division of HIV, Infectious Diseases, and Global Medicine, Department of Medicine, University of California San Francisco, San Francisco, California, USA; Division of HIV, Infectious Diseases, and Global Medicine, Department of Medicine, University of California San Francisco, San Francisco, California, USA; Division of Pulmonary, Critical Care, Allergy and Sleep Medicine, Department of Medicine, University of California San Francisco, San Francisco, California, USA; Infectious Diseases Research Collaboration, Kampala, Uganda; Infectious Diseases Research Collaboration, Kampala, Uganda; Infectious Diseases Research Collaboration, Kampala, Uganda; Division of Infection and Global Health, School of Medicine, University of St. Andrews, St. Andrews, UK; Department of Anesthesia and Critical Care, University of California San Francisco, San Francisco, California, USA; Division of HIV, Infectious Diseases, and Global Medicine, Department of Medicine, University of California San Francisco, San Francisco, California, USA; Division of HIV, Infectious Diseases, and Global Medicine, Department of Medicine, University of California San Francisco, San Francisco, California, USA; Infectious Diseases Research Collaboration, Kampala, Uganda; Division of Experimental Medicine, Department of Medicine, University of California San Francisco, San Francisco, California, USA; Division of Pulmonary Medicine, Department of Medicine, Makerere College of Health Sciences, Kampala, Uganda; Department of Epidemiology of Microbial Diseases, Yale School of Public Health, New Haven, Connecticut, USA; Pulmonary, Critical Care, and Sleep Medicine Section, Yale School of Medicine, New Haven, Connecticut, USA; Division of Pulmonary, Critical Care and Sleep, Department of Medicine, Veterans Affairs (VA) Puget Sound Health Care System and University of Washington, Seattle, Washington, USA; Division of Pulmonary Medicine, Department of Medicine, Makerere College of Health Sciences, Kampala, Uganda; Division of Pulmonary Medicine, Department of Medicine, Mulago Hospital and Complex, Kampala, Uganda; Division of HIV, Infectious Diseases, and Global Medicine, Department of Medicine, University of California San Francisco, San Francisco, California, USA; Division of Pulmonary, Critical Care, Allergy and Sleep Medicine, Department of Medicine, University of California San Francisco, San Francisco, California, USA

**Keywords:** HIV, preserved ratio impaired spirometry, sex differences, tuberculosis

## Abstract

**Background:**

Preserved ratio impaired spirometry (PRISm), defined as a normal ratio of forced expiratory volume in 1 second (FEV_1_) to forced vital capacity (≥0.70) with low FEV_1_ (<80% predicted), has been associated with increased mortality in the general population. Female sex has been associated with increased odds of PRISm in people without HIV. People with HIV (PWH) are at increased risk for lung function abnormalities, but whether HIV modifies the effect of sex on PRISm development is largely unknown.

**Methods:**

Adults with and without HIV underwent baseline followed by serial spirometry after completing therapy for pneumonia, predominantly tuberculosis (TB), in Kampala, Uganda. Using generalized estimating equations adjusted for age, body mass index, smoking, biomass fuel exposure, HIV, and TB status, we compared individuals with PRISm with those with normal spirometry. These models were stratified by HIV status.

**Results:**

Of 339 baseline participants, 153 (45%) were women; 129 (38%) had HIV, of whom 53% were women. Overall, 105/339 participants (31%) had PRISm at baseline. HIV was associated with lower odds of PRISm (adjusted odds ratio [aOR], 0.38; 95% CI, 0.21–0.68; *P* = .001). Female sex trended toward increased odds of PRISm among all participants (aOR, 1.65; 95% CI, 0.99–2.75; *P* = .052). The association between female sex and PRISm tended to be stronger among PWH (aOR, 3.16; 95% CI, 1.14–8.76; *P* = .03) than among those without HIV (aOR, 1.34; 95% CI, 0.73–2.45; *P* = .34); this study was underpowered to detect an HIV–sex interaction of this magnitude (*P* = .30).

**Conclusions:**

Among Ugandan adults who recovered from pneumonia, female sex was associated with increased odds and HIV with decreased odds of PRISm, suggesting independent sex and HIV effects on PRISm pathogenesis.

Chronic respiratory disease is a leading cause of death globally [1]. The most commonly diagnosed chronic lung disease is chronic obstructive pulmonary disease (COPD), which is characterized by airflow obstruction and diagnosed by a decreased ratio of forced expiratory volume in 1 second (FEV_1_) to forced vital capacity (FVC) on postbronchodilator spirometry [2]. Airflow obstruction serves as an important cause of both morbidity and mortality worldwide [2, 3].

Nonobstructive lung function abnormalities are increasingly being recognized as harbingers of mortality or respiratory disease–associated morbidity. Preserved ratio impaired spirometry (PRISm), which is defined as a normal FEV_1_/FVC ratio but with an abnormal FEV_1_, has been independently associated with increased mortality [4–6] and a higher respiratory symptom burden [7–9]. Patients with PRISm also demonstrate increased frequency in progression from normal FEV_1_/FVC ratio to airflow obstruction over time [7, 10, 11]. For example, a large UK-based study found an 11% prevalence of PRISm, with 12% of individuals with PRISm at baseline subsequently developing COPD over a median of 9 years of follow-up [7]. Studies in the general population have found that female sex is associated with increased odds of PRISm development [4, 7, 12]. Other risk factors include high and low body mass index (BMI), cigarette smoking, exposure to biomass fuel, and tuberculosis (TB) [12–15].

Women with HIV may be at particular risk for developing PRISm. A cross-sectional study in India evaluating lung function among people with HIV (PWH) demonstrated that female sex was independently associated with a 22-fold higher odds of having PRISm compared with male sex, even when controlling for TB status and biomass fuel exposure [16]. Notably, this cohort had low rates of airflow obstruction and did not find HIV-related factors, such as HIV viral load, to be significantly associated with the presence of abnormal lung function. In a Uganda-based cohort, longitudinal FEV_1_ decline was noted only among women with HIV, a finding that was not seen among women without HIV or among men with and without HIV [17]. While there are several potential explanations for these findings, such as sex serving as a biologic risk factor or sex serving as a confounder for unmeasured exposures, these findings suggest that both sex-specific drivers and HIV-associated factors may play a role in PRISm development.

To determine whether women with HIV in urban Uganda have higher odds of PRISm after acute pneumonia compared with men with and without HIV and women without HIV, we conducted a longitudinal study among Ugandan adults evaluating the associations of HIV and sex with PRISm, FEV_1_, and FVC overall, and then subsequently stratified by HIV status. Our primary objective was to investigate whether sex-based differences in PRISm prevalence exist when compared with normal spirometry among PWH after acute pneumonia, predominantly pulmonary TB. Given the recommendation to use the lower limit of normal (LLN) for the diagnosis of abnormal lung function by the American Thoracic Society/European Respiratory Society (ATS/ERS) [18], we then performed a sensitivity analysis using the LLN as the cutoff value for PRISm, FEV_1_, and FVC.

## METHODS

### The I AM OLD Study

The Inflammation, Aging, Microbes, and Obstructive Lung Disease (I AM OLD) Study is a prospective, longitudinal cohort study that enrolls participants in the United States (San Francisco and Seattle) and Kampala, Uganda. The study evaluates the development and progression of lung function abnormalities in adults with and without HIV after resolution of acute pneumonia. After enrolling participants at the time of acute pneumonia, the study performs longitudinal lung function testing and collects serial immune activation and telomere biology markers to elucidate potential mechanistic insights into lung function abnormalities among PWH. This analysis focuses on participants enrolled in Uganda.

### Uganda Cohort

We enroll nonpregnant patients aged 18–60 at the time of acute pneumonia, including pulmonary TB. Participants are enrolled from the inpatient wards or the outpatient TB clinic if they have presented with suspected pneumonia at the China-Uganda Friendship Hospital Naguru in Kampala, Uganda. Patients are eligible for inclusion if they have had a cough for <6 months. All participants are tested for HIV at enrollment and at each follow-up visit. For participants with HIV, CD4 counts are checked and antiretroviral therapy status is assessed, but HIV RNA levels are not available.

After enrollment, participants receive a chest radiograph and undergo protocolized evaluation for pulmonary TB, as described previously [19–21]. If TB-positive, participants receive TB therapy followed by baseline post-treatment pre- and postbronchodilator spirometry after completion of TB therapy. Participants then receive follow-up spirometry 3 months after the baseline visit to assess the stability of the spirometry results in the immediate post-TB period. If TB testing is negative, participants are treated for pneumonia followed by baseline post-treatment pre- and postbronchodilator spirometry at least 3 months after therapy completion. All participants continue to undergo pre- and postbronchodilator spirometry testing annually.

### Ethical Approvals

Ethical approval was obtained from the Makerere University School of Medicine Research and Ethics Committee (REC REF No. 2006-017) and the University of California San Francisco Institutional Review Board (IRB #10-02633). All study participants were informed about the study by a nurse or physician and provided written informed consent before participation in the study.

### Study Population and Procedures

This current secondary longitudinal analysis from Uganda spans visits from February 2016 to October 2022. Pre- and postbronchodilator spirometry was performed by trained study personnel using an Easy on-PC (February 2016–October 2021) or an EasyOne Pro device (October 2021–October 2022; ndd Medizintechnik AG, Zurich, Switzerland) according to ATS/ERS guidelines [18, 22]. Each spirometry maneuver was over-read by a trained respiratory therapist to confirm acceptability and reproducibility of test results. Using the ATS/ERS spirometry quality grading system, tests with grades A through C were included in the analysis [18, 22].

### Classification of Lung Function Patterns

PRISm was defined as a postbronchodilator FEV_1_/FVC ratio ≥0.70 and a postbronchodilator FEV_1_ <80% predicted (the fixed ratio definition), as defined in prior studies [4, 5, 10]. An abnormal FVC was defined as <80% predicted. Normal spirometry was defined as a postbronchodilator FEV_1_/FVC ratio ≥0.70 with an FEV_1_ and FVC both ≥80% predicted. In recognition of the ATS/ERS guidelines, a sensitivity analysis was performed with PRISm defined as FEV_1_/FVC ratio ≥ LLN with an FEV_1_ < LLN (the LLN definition) [18]. Normal spirometry was defined as FEV_1_/FVC ratio ≥ LLN with an FEV_1_ and FVC both ≥ LLN [18]. Participants with COPD or spirometric restriction were not included in the analysis to avoid heterogeneity between comparison groups and potential overlap of PRISm with spirometric restriction. The Global Lung Initiative African American equations were used for both the fixed ratio and LLN definitions, per the ATS/ERS guidelines [18, 23].

### Statistical Analysis

Participants included in this analysis had acceptable spirometry results and known TB status. In this analysis, time-independent variables were used at the time of initial enrollment with pneumonia, with age, BMI, and smoking status used at each respective visit. Tertiles were used for BMI as the models would not converge when using clinical BMI cutoffs due to few obese participants in the cohort, and trend with increasing BMI was assessed. Demographics and clinical characteristics were compared between those with PRISm and those with normal spirometry. Multivariable analyses were performed at the baseline spirometry visit examining the associations between demographic parameters, including sex, age, and BMI, as well as important clinical factors, including ever smoking status (self-report yes/no), biomass fuel exposure (self-report yes/no), HIV status, and TB status with PRISm, FEV_1_ <80% predicted, and FVC <80% predicted. A sensitivity analysis was performed using the LLN definitions for PRISm, FEV_1_, and FVC. These models were then stratified by HIV status to examine whether the associations differed among people with and without HIV. Generalized estimating equations (GEEs) with a log link and binomial distribution were subsequently used across all spirometry visits, controlling for biologically important predictors (age, BMI, smoking status, biomass fuel exposure, HIV, and TB status). Repeated measures were used at each available spirometry visit (ie, baseline and subsequent visits) for participants with either PRISm or normal spirometry without censoring. All observations where a participant's spirometry demonstrated PRISm or was classified as normal were included in the analysis. As using age as a continuous variable led to convergence issues in the GEE models, age was dichotomized to the sample median. The models were stratified by HIV status due to biologic plausibility and our a priori study question. As there were only 13 participants with HIV who did not have TB, TB status was excluded from the stratified model.

## RESULTS

### Baseline Participant Characteristics

**Figure 1.**
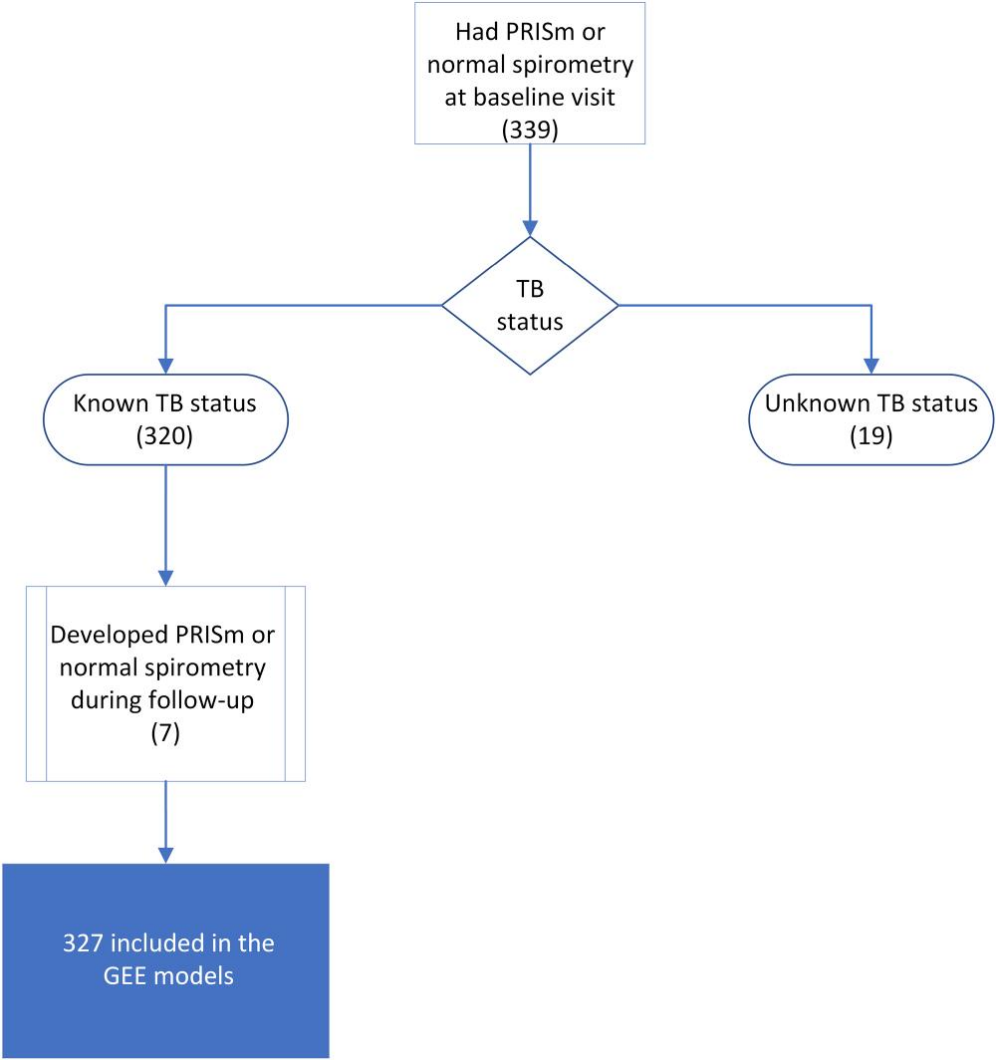
Flow diagram of participant inclusion in the analysis. Abbreviations: GEE, generalized estimating equation; PRISm, preserved ratio impaired spirometry; TB, tuberculosis.

Overall, 1013 participants were enrolled in I AM OLD during an episode of acute pneumonia. Of the 392 participants with passing spirometry, 339 had PRISm or normal spirometry at baseline (Figure 1). There were 43 participants with known TB status who never had PRISm or normal spirometry and were therefore excluded from the longitudinal analysis. Of those 43 participants, 22 were PWH, of whom 19 had obstruction and 3 had spirometric restriction. Of the 339 included baseline participants, 153 (45%) were women and 129 (38%) were PWH (Table 1). Among those with HIV, 68 (53%) were women. Of those who were lost to follow-up, 322 had HIV (54%) as compared with 271 (46%) without HIV. However, the proportion of women who were lost to follow-up was similar to those included in the analysis (48% of those lost to follow-up were female vs 53% included in the analysis).

**Table 1.** Participant Characteristics Among Those With and Without PRISm at Baseline

	Fixed Ratio Definition			Lower Limit of Normal Definition		
	PRISm^a^ No. (%) or Median (IQR) n = 105	Normal^a^ No. (%) or Median (IQR) n = 234	*P* Value	PRISm^b^ No. (%) or Median (IQR) n = 81	Normal^b^ No. (%) or Median (IQR) n = 242	*P* Value
Age, y	29 (23–38)	32 (27–41)	**.005**	31 (23–38)	32 (27–41)	**.01**
Female sex	49 (47)	104 (44)	.72	37 (46)	107 (44)	.89
BMI, kg/m^2^	20.0 (18.7–21.9)	21.6 (20.0–24.0)	**<.001**	19.6 (18.5–21.5)	21.5 (19.8–24.0)	**<.001**
Ever cigarette smoker	22 (21)	38 (16)	.36	19 (23)	43 (18)	.25
Exposure to biomass fuel at home	76 (74)	168 (73)	.89	58 (73)	175 (73)	.89
HIV status	…	…	**<.001**	**…**	**…**	**<.001**
Positive	26 (25)	103 (44)	**…**	18 (22)	104 (43)	**…**
Negative	79 (75)	131 (56)	**…**	63 (78)	138 (57)	**…**
CD4 count^a^	150 (65–297)	112 (32–293)	.47	143 (65–313)	120 (35–303)	.50
CD4 <200 cells/mm^3^	7 (33)	27 (35)	.90	3 (21)	27 (35)	.37
CD4 ≥200 cells/mm^3^	14 (67)	50 (65)	**…**	11 (79)	50 (65)	**…**
Current ART use^a^	14 (54)	40 (39)	**.03**	9 (50)	41 (20)	**.03**
TB status	…	…	.67	…	…	.42
Positive TB status	86 (82)	185 (79)	**…**	69 (85)	188 (78)	**…**
Negative TB status	15 (14)	34 (15)	**…**	9 (11)	39 (16)	**…**
Unknown TB status	4 (4)	15 (6)	**…**	3 (4)	15 (6)	**…**

The bold values are those that reached statistical significance.

Abbreviations: BMI, body mass index; FEV_1_%predicted, forced expiratory volume in 1 second as percentage of predicted reference value; FEV_1_/FVC ratio, ratio of forced expiratory volume in 1 second to forced vital capacity; FVC% predicted, forced vital capacity as percentage of predicted reference value; IQR, interquartile range; PRISm, preserved ratio impaired spirometry; TB, tuberculosis.

^a^PRISm defined as FEV_1_/FVC ≥0.70 and FEV_1_ <80% predicted, and normal defined as FEV_1_/FVC ≥0.70 and FEV_1_ ≥80% predicted and FVC ≥80% predicted.

^b^PRISm defined as FEV_1_/FVC ≥ LLN and FEV_1_ < LLN, and normal defined as FEV_1_/FVC ≥ LLN and FEV_1_ ≥ LLN and FVC ≥ LLN.

^c^Among those with HIV.

At baseline, 105 (31%) individuals had PRISm and 234 (69%) had normal spirometry using the fixed ratio definitions for FEV_1_/FVC and FEV_1_. Demographic characteristics were similar between the PRISm and normal spirometry groups, except that those with normal spirometry were slightly older (median, 32 vs 29 years; *P* = .005) and had a slightly higher median BMI (median, 21.6 vs 20.0 kg/m^2^; *P* < .001). Prevalence of cigarette smoking and biomass fuel exposure were similar between groups. There was a higher proportion of PWH in the normal spirometry group than among those with PRISm (44% vs 25%; *P* < .001), but among those with HIV, those with PRISm were more likely to be on ART (54% vs 39%; *P* = .03). The median time between ART initiation and the baseline visit (interquartile range) was 177 (155–201) days. TB positivity was similar between groups (82% with PRISm vs 79% with normal spirometry). When the LLN was used as the cutoff definition, the demographic characteristics were similar between the PRISm and normal spirometry groups and mirrored those of the fixed ratio definition (Table 1).

### Longitudinal Associations of Participant Characteristics With PRISm

Of the 339 baseline participants, 19 individuals had unknown or indeterminate TB status at baseline and were excluded from the GEE models (Figure 1). Seven individuals who had initially been excluded at the baseline visit subsequently developed either normal spirometry or PRISm during follow-up and were included in the longitudinal analysis, leading to a total of 327 participants across 560 visits included in the GEE models (Table 2). Overall, female sex trended toward increased odds of PRISm in the adjusted analysis (adjusted odds ratio [aOR], 1.65; 95% CI, 0.99–2.75; *P* = .052). In both the unadjusted and adjusted models, HIV was associated with lower odds of PRISm (aOR, 0.38; 95% CI, 0.21–0.68; *P* = .001). The study was underpowered to detect an HIV–sex interaction of this magnitude (*P* = .30).

**Table 2.** The Longitudinal Adjusted Associations of Participant Characteristics With PRISm Using the Fixed Ratio Definition^b^

…	Overall (n_participants_ = 327; n_obs_ = 560)				HIV+ (n_participants_ = 120; n_obs_ = 211)		HIV- (n_participants_ = 207; n_obs_ = 349)	
	Unadjusted PRISm vs Normal OR (95% CI)	*P* Value	Adjusted PRISm^a^ vs Normal aOR (95% CI)	*P* Value	PRISm^a^ vs Normal aOR (95% CI)	*P* Value	PRISm^a^ vs Normal aOR (95% CI)	*P* Value
Age >32 y	0.83 (0.51–1.34)	.45	1.23 (0.71–2.14)	.45	1.59 (0.46–5.55)	.46	1.05 (0.59–1.87)	.89
Female sex	1.19 (0.77–1.85)	.43	1.65 (0.99–2.75)	.052	**3.16 (1.14–8.76)**	**.03**	1.34 (0.73–2.45)	.34
BMI, tertile 2 vs 1^c^	**0.62 (0.39–0.99)**	**.046**	**0.55 (0.34–0.89)**	**.01**	**0.23 (0.07–0.76)**	**.02**	0.73 (0.45–1.19)	.21
BMI, tertile 3 vs 1	**0.35 (0.20–0.61)**	**<.001**	**0.29 (0.16–0.53)**	**<.001**	**0.21 (0.07–0.63)**	**.005**	**0.34** (0**.17–0.68)**	.**002**
*P*_trend_	…	**<.001**	…	**<.001**	…	**.05**	…	.**03**
Ever cigarette smoker	1.43 (0.79–2.57)	.24	1.27 (0.62–2.55)	.51	1.23 (0.36–4.21)	.74	1.27 (0.55–2.92)	.57
Exposure to biomass fuel at home	0.99 (0.59–1.68)	.99	0.86 (0.48–1.53)	.61	0.52 (0.16–1.78)	.30	0.96 (0.50–1.86)	.91
HIV+ vs HIV-	**0.48 (0.29–0.80)**	**.005**	**0.38 (0.21–0.68)**	**.001**	-	-	-	-
TB+ vs TB-	0.92 (0.48–1.75)	.80	0.55 (0.27–1.10)	.10	-	-	-	-

Abbreviations: aOR, adjusted odds ratio; BMI, body mass index; FEV_1_%predicted, forced expiratory volume in 1 second as percentage of predicted reference value; FEV_1_/FVC ratio, ratio of forced expiratory volume in 1 second to forced vital capacity; FVC% predicted, forced vital capacity as percentage of predicted reference value; IQR, interquartile range; OR, odds ratio; PRISm, preserved ratio impaired spirometry; TB, tuberculosis.

n_participants_ = the number of participants included in the analysis; n_obs_ = the number of unique observations included in the analysis. The bold values are those that reached statistical significance.

^a^PRISm defined as FEV_1_/FVC ≥0.70 and FEV_1_ <80% predicted, and normal defined as FEV_1_/FVC ≥0.70 and FEV_1_ ≥80% predicted and FVC ≥80% predicted.

^b^Adjusted for age, BMI, smoking status, biomass fuel exposure, HIV, and TB status.

^c^BMI was divided into tertiles to facilitate model convergence. For clinical interpretation, trend was also assessed.

### Longitudinal Associations of Sex With PRISm Stratified by HIV Status

In the adjusted stratified analysis among those with HIV, women had 3.16-fold higher adjusted odds of PRISm than men (95% CI, 1.14–8.76; *P* = .03) (Table 2) using the fixed ratio definition. Among those without HIV, however, there was no evidence of an association between female sex and PRISm (aOR, 1.34, 95% CI, 0.73–2.45; *P* = .34). When time was included in the models, the inferences were similar. Overall, women had non–statistically significantly higher odds of PRISm (aOR, 1.56; 95% CI, 0.90–2.70; *P* = .11), whereas women with HIV had 3.62 higher odds of PRISm compared with normal spirometry (95% CI, 1.32–9.90; *P* = .01, results not shown). Women without HIV did not demonstrate higher odds of PRISm (aOR, 1.10).

**Figure 2.**
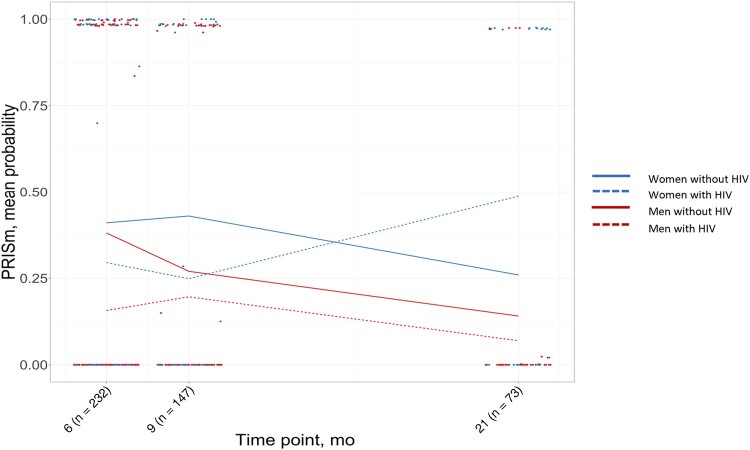
Mean probability of PRISm by HIV status and sex over time. Abbeviation: PRISm, preserved ratio impaired spirometry.

In Figure 2, the predicted probabilities of PRISm for women and men with and without HIV as a function of time using the models described above are pictured. Over time, men with and without HIV have a lower mean probability of developing PRISm. Women without HIV start with a higher mean probability of PRISm than women with HIV as well as compared with men with and without HIV, but the mean probability decreases over time. In contrast, women with HIV start with a lower mean probability for PRISm at baseline but develop a higher mean probability for PRISm than all other groups by month 21.

### Longitudinal Associations of Sex With Abnormal FEV_1_ and FVC Stratified by HIV Status

When evaluating the overall presence of abnormal FEV_1_ during follow-up, female sex was associated with 1.68 higher odds of having an FEV_1_ <80% predicted (95% CI, 1.05–2.69; *P* = .03) (Table 3). As expected from the PRISm results, HIV was associated with lower odds of having an FEV_1_ <80% predicted (aOR, 0.50; 95% CI, 0.30–0.83; *P* = .01).

**Table 3.** The Longitudinal Adjusted Associations of Participant Characteristics With FEV_1_ <80% Predicted and FVC <80% Predicted Using the Fixed Ratio Definition^a^

Overall	FEV_1_ <80% Predicted vs Normal aOR (95% CI)	*P* Value	FVC <80% Predicted vs Normal aOR (95% CI)	*P* Value
	n_participants_ = 358; n_obs_ = 617		n_participants_ = 341; n_obs_ = 575	
Age >32 y	1.34 (0.85–2.11)	.21	1.16 (0.72–1.89)	.54
Female sex	**1.68 (1.05–2.69)**	**.03**	1.53 (0.93–2.50)	.09
BMI, tertile 2 vs 1^b^	**0.60 (0.41–0.87)**	**.01**	**0.48 (0.33–0.69)**	**<.001**
BMI, tertile 3 vs 1	**0.33 (0.21–0.54)**	**<.001**	**0.25 (0.16–0.41)**	**<.001**
*P*_trend_	…	**<.001**	…	**<.001**
Ever cigarette smoker	1.49 (0.82–2.72)	.19	1.32 (0.69–2.49)	.40
Exposure to biomass fuel at home	0.87 (0.51–1.47)	.59	0.75 (0.44–1.29)	.31
HIV+ vs HIV-	**0.50 (0.30–0.83)**	**.01**	**0.58 (0.33–0.94)**	**.03**
TB+ vs TB-	0.54 (0.29–1.00)	.05	0.75 (0.38–1.49)	.41
**HIV+**	n_participants_ = 137; n_obs_ = 239		n_participants_ = 130; n_obs_ = 221	
Female sex	**3.20 (1.39–7.36)**	**.006**	**3.20 (1.26–8.13)**	**.02**
**HIV-**	n_participants_ = 221; n_obs_ = 378		n_participants_ = 211; n_obs_ = 354	
Female sex	1.26 (0.72–2.23)	.42	1.23 (0.68–2.25)	.49

n_participants_ = the number of participants included in the analysis; n_obs_ = the number of unique observations included in the analysis. The bold values are those that reached statistical significance.

Abbreviations: aOR, adjusted odds ratio; BMI, body mass index; FEV_1_%predicted, forced expiratory volume in 1 second as percentage of predicted reference value; FVC% predicted, forced vital capacity as percentage of predicted reference value; TB, tuberculosis.

^a^Adjusted for age, BMI, smoking status, biomass fuel exposure, HIV, and TB status.

^b^BMI was divided into tertiles to facilitate model convergence. For clinical interpretation, trend was also assessed.

In the stratified analysis, among those with HIV, women had 3.20 times higher odds of having a low FEV_1_ than men with HIV (95% CI, 1.39–7.36; *P* = .006); women without HIV did not demonstrate higher odds of an abnormal FEV_1_ when compared with their male counterparts. Similar patterns were seen for those with an FVC <80% predicted over follow-up (Table 3).

### Longitudinal Associations of Sex and Abnormal Lung Function Using the Lower Limit of Normal

In the models evaluating the associations of participant characteristics during follow-up with PRISm using the LLN definition, female sex was associated with 1.88 higher odds of PRISm (95% CI, 1.09–3.24; *P* = .02) (Supplementary Table 1). In the stratified model, among those with HIV, female sex was non–statistically significantly associated with increased odds of PRISm (aOR, 1.77; 95% CI, 0.66–4.71; *P* = .26). Women without HIV had similar non–statistically significant associations of increased odds of PRISm (aOR, 1.71; 95% CI, 0.90–3.23; *P* = .10). Using the LLN as the cutoff value, similar inferences were observed with FEV_1_ and FVC during follow-up (Supplementary Table 2).

## DISCUSSION

In this longitudinal cohort of adults who recovered from pneumonia in Kampala, Uganda, the odds of PRISm among women with HIV were up to 3 times higher than the odds for men with HIV. Similar to prior studies, there was an overall association of female sex with PRISm [4, 7]. Overall, 31% of participants in this selected cohort had PRISm at baseline using the fixed ratio definition, whereas 25% of participants had PRISm at baseline when using the LLN definition. Regardless of the definition used, HIV was associated with lower odds of PRISm development. Participants with HIV demonstrated a >60% reduction in odds of PRISm compared with their seronegative counterparts. Taken together, these findings suggest that both sex and HIV-associated factors may contribute to PRISm development.

Our findings of a sex difference in HIV are similar to but of a smaller magnitude than a study by Baidya et al. of women (n = 121) and men (n = 89) with HIV [16]. This study found that female sex was independently associated with 22-fold higher odds of having PRISm when compared with males, even after adjusting for BMI, smoking, diabetes, TB status, and HIV-associated factors (95% CI, 7.42–69.92; *P* < .001). Similar to our analysis, female sex was also associated with lower FEV_1_ and FVC. Differences in the magnitude of our results could be attributed to differences in our cohort, which is younger (median age, 32 vs 40) and has a higher burden of tuberculosis (∼80% as compared with 40%), perhaps leading to higher rates of post-tuberculosis airflow obstruction or spirometric restriction. Furthermore, in the Baidya cohort, 46% of participants were categorized as having PRISm, whereas only 3% had airflow obstruction as compared with 19% with airflow obstruction among those with HIV in our cohort. Despite these differences, the findings from the Baidya study are in the same direction as observed in this analysis and in the general population, in which female sex has been identified as a risk factor for PRISm development [4, 7].

Among those with HIV, women demonstrated increased odds for PRISm development as well as a low FEV_1_ and FVC, with women with HIV demonstrating statistically significantly higher odds of PRISm when using the fixed ratio definition for FEV_1_/FVC. When using the LLN as the cutoff, overall, female sex was associated with higher odds of PRISm, but an HIV-associated difference was not seen in the stratified analysis. While the seminal papers by Wan et al. define PRISm using the fixed definition (a fixed ratio of FEV_1_/FVC ≥0.70 with an FEV_1_ <80% predicted) [4, 10, 24], the ATS/ERS recommends use of the LLN for the diagnosis of airflow obstruction given that changes in lung function over time are dependent on many factors, including age and sex [18]. Due to sex differences in the dysanaptic growth of the airways as compared with the lungs, the FEV_1_/FVC ratio is typically higher in women [25]. While it is possible that using the LLN was therefore more accurate in our cohort, thereby demonstrating that female sex alone is associated with higher odds of PRISm, as has been seen in prior studies [4, 7, 12], this is belied by the FEV_1_ results. Specifically, we found that women with HIV uniquely demonstrated significantly higher odds of an abnormal FEV_1_ under both definitions. Prior studies have also demonstrated an HIV-specific, sex-specific FEV_1_ decline. A study by McNeill et al. conducted in a rural Ugandan cohort (n = 278) with low rates of TB similarly found an accelerated rate of decline in FEV_1_ over time among women with HIV as compared with women without HIV, a finding that was not seen among men with and without HIV [17]. These findings suggest that despite differences in odds of PRISm depending on the cutoff definition used, sex-specific and HIV-specific factors appear to play a role in the development of abnormal lung function, particularly FEV_1_ decline.

Despite PWH being at higher risk for COPD [26–29], there have been few data on rates of nonobstructive lung function abnormalities, such as PRISm, among PWH. Chronic lung disease represents the second most common comorbidity among PWH [26]; HIV infection is independently associated with COPD, even when controlling for cigarette smoking [28, 30]. PWH have also been found to have an earlier onset and a more rapid decline in lung function than the general population [28, 31, 32]. Interestingly, in our analysis, we found that HIV was associated with lower odds of PRISm when compared with normal spirometry. This is in contrast to a cross-sectional analysis in a Nigerian cohort of adults with and without HIV by Fink et al. in which there was no HIV-associated difference in PRISm prevalence [33]. Notably, in the Fink analysis, a history of TB was significantly higher among those with HIV when compared with those without HIV (28.8% vs 0.7%), and the median FEV_1_ and FVC were both lower among PWH than among their seronegative counterparts. Given the parent study's design, our participants were recruited after an episode of acute pneumonia, whereas the participants in the Fink study were recruited from outpatient clinics. Our discrepant results may also be related to selection bias as participants with PRISm were compared with those with normal spirometry alone, with participants with HIV exhibiting a higher prevalence of abnormal lung function than their seronegative counterparts. For example, the prevalence of obstructive lung disease in this cohort was slightly higher in PWH (19%, or 21 individuals) as compared with those without HIV (13%, or 18 individuals), suggesting that the association of HIV with lower odds of PRISm in this analysis could be due to higher rates of HIV-associated COPD or spirometric restriction. In addition, different definitions were used in the Fink study, which may have contributed to our different results. In the Fink study, the LLN was used for FEV_1_, FVC, and their ratio, whereas PRISm was defined using the fixed cutoff definition, allowing for overlap between PRISm and other lung function abnormalities.

Our study has several limitations. As our participants underwent spirometry after recovery from pneumonia, it is uncertain whether these results are generalizable to PWH without recent pneumonia. There were overall low numbers of TB-negative participants given our enrollment strategy, thereby reducing the power of the analysis to evaluate the association of non-TB pneumonia with abnormal lung function. We were similarly underpowered to detect an HIV–sex interaction in this study. While we had longitudinal lung function testing, many of our enrolled participants were lost to follow-up, and most of our participants did not have pulmonary function testing beyond 9 months of follow-up at the time of this analysis, limiting our ability to determine the durability of PRISm and its potential progression to COPD over time. We do not have the amount of biomass fuel exposure or cigarette smoking intensity as part of our data collection, which may have introduced measurement error into our analysis. Additional data to better evaluate socioeconomic status were not collected, and, among women, menopausal status was not determined. In the general population, early menopause has been associated with lower FEV_1_/FVC [34], and a prior study found that women with amenorrhea for >6 months had lower FEV_1_ and FVC than women with a normal menstrual flow [35]. Longitudinal lung function is currently being measured, and additional metrics on biomass fuel exposure, socioeconomic factors, and menstrual status data among female participants are currently being collected.

The strengths of this study include the nature of the cohort, which includes participants both with and without HIV and is highly enriched with women, which parallels the epidemiology of HIV in Uganda and in Sub-Saharan Africa. To date, there have been no studies evaluating the relationship between biologic sex and the development of PRISm among women with HIV despite the association of PRISm with increased mortality [4–6]. Evidence suggests that women with HIV are at higher risk for developing aging-related comorbidities compared with men [36, 37]; the progression and development of lung function abnormalities within this group merit further study.

## CONCLUSIONS

Among Ugandans with HIV who have recovered from pneumonia, female sex increases the odds of PRISm while HIV decreases the odds of PRISm, consistent with potential independent sex and HIV effects on PRISm pathogenesis. Given the known increased mortality associated with PRISm, studies to elucidate underlying mechanistic pathways are warranted.

## Supplementary Material

ofae033_Supplementary_Data

## Supplementary Data

Supplementary materials are available at *Open Forum Infectious Diseases* online. Consisting of data provided by the authors to benefit the reader, the posted materials are not copyedited and are the sole responsibility of the authors, so questions or comments should be addressed to the corresponding author.
