# Supplementary material for: Sex and HIV Differences in Preserved Ratio Impaired Spirometry (PRISm) Among Ugandans Postpneumonia
Source: Open Forum Infect Dis. 2024 Jan 22;11(3):ofae033. doi: 10.1093/ofid/ofae033 (PMC10919919; doi:10.1093/ofid/ofae033)
Supplement: ofae033_Supplementary_Data [file ofae033_supplementary_data.pdf]

Supplementary Table 1. The longitudinal adjusted associations of participant characteristics with PRISm by LLN*

	Overall ($N_{participants}$ =317; N_{obs} =541)				HIV+ ($N_{participants}$ =117; N_{obs} =203)		HIV- ($N_{participants}$ =200; N_{obs} =338)	
	**PRISm vs normal OR (95% CI)**	**P-value**	**PRISm† vs normal aOR (95% CI)**	**P-value**	**PRISm† vs normal aOR (95% CI)**	**P-value**	**PRISm† vs normal aOR (95% CI)**	**P-value**
Age, > 32 years	1.02 (0.63, 1.66)	0.93	1.37 (0.74, 2.53)	0.32	2.19 (0.35, 13.6)	0.40	1.10 (0.58, 2.05)	0.78
Female sex	1.27 (0.79, 2.05)	0.32	**1.88 (1.09, 3.24)**	**0.02**	1.77 (0.66, 4.71)	0.26	1.71 (0.90, 3.23)	0.10
BMI, tertile 2 vs 1[1]	0.65 (0.42, 1.01)	0.06	0.63 (0.39, 1.01)	0.05	**0.55 (0.31, 0.98)**	**0.04**	0.66 (0.39, 1.09)	0.10
BMI, tertile 3 vs 1	**0.37 (0.20, 0.68)**	**0.001**	**0.32 (0.17, 0.63)**	**<0.001**	**0.44 (0.22, 0.88)**	**0.02**	**0.31 (0.15, 0.64)**	**0.001**
Trend		**0.001**		**<0.001**		**0.01**		**<0.001**
Ever cigarette smoker	1.54 (0.83, 2.85)	0.17	1.43 (0.68, 3.02)	0.35	1.60 (0.43, 6.01)	0.48	1.31 (0.54, 3.16)	0.55
Exposure to biomass fuel at home	1.02 (0.59, 1.79)	0.94	0.90 (0.50, 1.63)	0.73	0.96 (0.28, 3.28)	0.95	0.93 (0.46, 1.83)	0.82
HIV+ vs HIV-	**0.43 (0.24, 0.76)**	**0.004**	**0.33 (0.18, 0.66)**	**<0.001**	-	-	-	-
TB+ vs TB-	1.16 (0.58, 2.32)	0.69	0.65 (0.30, 1.41)	0.28	-	-	-	-

† PRISm defined as FEV1/FVC ≥ LLN and FEV1 < LLN and normal defined as FEV1/FVC ≥ LLN and FEV1 ≥ LLN and FVC ≥ LLN

*Adjusted for age, BMI, smoking status, biomass fuel exposure, HIV, and TB status

[1]BMI was divided into tertiles to facilitate model convergence. For clinical interpretation, trend was also assessed.

$N_{participants}$ = the number of participants included in the analysis; N_{obs} = the number of unique observations included in the analysis

Supplementary Table 2. The longitudinal adjusted associations of participant characteristics with FEV_1 <LLN and FVC <LLN*

	**FEV_1 < LLN vs normal aOR (95% CI)**	**P-value**	**FVC < LLN vs normal aOR (95% CI)**	**P-value**
**Overall**	**$N_{participants}$ = 355; N_{obs} = 608**		**$N_{participants}$ =335; N_{obs} =564**	
Age, > 32 years	1.30 (0.82, 2.06)	0.26	1.15 (0.68, 1.91)	0.59
Female sex	**1.81 (1.11, 2.94)**	**0.02**	1.53 (0.91, 2.56)	0.11
BMI, tertile 2 vs 1[1]	**0.66 (0.48, 0.92)**	**0.01**	**0.42 (0.29, 0.61)**	**<0.001**
BMI, tertile 3 vs 1	**0.36 (0.23, 0.57)**	**<0.001**	**0.20 (0.12, 0.34)**	**<0.001**
Trend		**<0.001**		**<0.001**
Ever cigarette smoker	1.59 (0.89, 2.90)	0.13	0.95 (0.48, 1.86)	0.88
Exposure to biomass fuel at home	0.97 (0.57, 1.63)	0.89	0.79 (0.46, 1.38)	0.41
HIV+ vs HIV-	**0.48 (0.29, 0.81)**	**0.006**	**0.55 (0.32, 0.94)**	**0.03**
TB+ vs TB-	0.67 (0.36, 1.27)	0.22	0.82 (0.41, 1.61)	0.56
**HIV+**	**$N_{participants}$ =134; N_{obs} =232**		**$N_{participants}$ =126; N_{obs} =216**	
Female sex	**2.83 (1.21, 6.62)**	**0.02**	**3.04 (1.17, 7.92)**	**0.02**
**HIV-**	**$N_{participants}$ =221; N_{obs} = 376**		**$N_{participants}$ =209; N_{obs} =348**	
Female sex	1.53 (0.86, 2.73)	0.15	1.23 (0.66, 2.31)	0.52

* Adjusted for age, BMI, smoking status, biomass fuel exposure, HIV, and TB status

[1]BMI was divided into tertiles to facilitate model convergence. For clinical interpretation, trend was also assessed.

$N_{participants}$ = the number of participants included in the analysis; N_{obs} = the number of unique observations included in the analysis
